# Genome-wide association study and transcriptome analysis reveal candidate genes related to drought stress in the germination stage of soybean

**DOI:** 10.3389/fpls.2025.1621869

**Published:** 2025-07-30

**Authors:** Keke Kong, Mengge Xu, Lanhua Wu, Huiwen Zhou, Ruikai Wang, Tuanjie Zhao, Chune Wang, Yingpei Song

**Affiliations:** ^1^ Soybean Research Institute, Institute of Jiangxi Oil-tea Camellia & College of Pharmacy and Life Science, Jiujiang University, Jiujiang, Jiangxi, China; ^2^ Key Laboratory of Biology and Genetics Improvement of Soybean, Ministry of Agriculture/Zhongshan Biological Breeding Laboratory (ZSBBL)/National Innovation Platform for Soybean Breeding and Industry-Education Integration/State Key Laboratory of Crop Genetics & Germplasm Enhancement and Utilization, College of Agriculture, Nanjing Agricultural University, Nanjing, China

**Keywords:** soybean, drought tolerance, germination stage, RTM-GWAS, RNA-seq

## Abstract

Drought is the major abiotic stress threatening soybean production globally. However, the genetic basis of soybean drought resistance at the germination stage remains largely unknown. In this study, the drought-tolerance phenotypes of a diverse panel of 207 soybean accessions were examined. Leveraging 95,043 high-density single-nucleotide polymorphism (SNP) markers, a total of 58 quantitative trait loci (QTLs) were detected using the restricted two-stage multi-locus genome-wide association study (RTM-GWAS) method, and 10 of these QTLs were considered as large-contribution QTLs that have larger phenotype variation. Furthermore, RNA-sequencing was performed on the roots of two soybean accessions with contrasting drought tolerance. A total of 1,183, 1,354, and 1,581 differentially expressed genes (DEGs) between two soybean accessions after 0h, 12h, and 24h of drought treatment were separately obtained, and 4,012 and 4,586 genes responsive to drought stress were identified at 12h and 24h, respectively. By utilizing these DEGs, a weighted gene co-expression network analysis (WGCNA) was constructed, and 13 distinct modules were obtained, among which four modules were considered as key modules. Subsequently, 40 hub genes were identified in these four modules. In addition, by combining RTM-GWAS and transcriptome analysis, 22 candidate genes underlying large-contribution QTLs were identified. Based on the functional annotations, *Glyma.12G141700*, *Glyma.15G040000*, *Glyma.05G049300*, *Glyma.14G105900*, and *Glyma.15G041100* were regarded as the most possible candidate genes that regulate soybean drought tolerance at the germination stage. The QTLs, key modules, and hub genes discovered in this study will provide a new understanding of the genetic basis of soybean drought resistance at the germination stage and lay a theoretical foundation for the improvement and innovation of high-quality soybean germplasm.

## 1 Introduction

Drought stress is a global ecological problem that seriously affected plant growth and development and decreased agricultural production (Du et al., 2009; Vijay et al., 2018). Under high drought conditions, it will cause various damages to the morphology and physiology of soybeans, such as seed germination, seedling development (Zia et al., 2021; Poudel et al., 2023), leaf wilting, and leaf ion imbalances (Jiang et al., 2024). Seed germination is the first stage for the soybean growth cycle. The overall number of seedlings may plummet by over 20% when drought stresses occur at the seed germination stage, which makes a great threat to soybean yield and food security (Devi et al., 2014; Zhao et al., 2017; Arya et al., 2021; Cotrim et al., 2021). Moreover, with increasing scarcity of water resources, irrigation is not a feasible option for most of the soybean-growing regions. Thus, understanding the genetic basis of drought tolerance at the germination stage is critical for breeders to develop drought-tolerant soybean cultivars in order to sustain higher yields and global food security under the prevailing climate change scenario.

Drought tolerance in soybean is a complex quantitative trait controlled by numerous genetic and nongenetic factors (Du et al., 2009; Dhanapal et al., 2015). Many QTLs controlling drought tolerance had been identified at soybean seedling or mature stages (Hwang et al., 2015; Ye et al., 2020). Recently, at the seeding stage, 33 drought-tolerance QTLs associated with wilting score, days-to-wilting, leaf relative water content, and leaf relative conductivity were identified in recombinant inbred lines derived from a cross between Lin (drought-sensitive variety) and Meng (drought-tolerant variety) (Jiang et al., 2024). In contrast, only a few studies had been concentrated on soybean drought tolerance during the germination stage. For example, in 259 released Chinese soybean cultivars, 4,616 SNPs were obtained, and 15 SNP loci associated with drought-tolerance indices were identified by genome-wide association studies (GWAS) (Liu et al., 2020). A total of 92 SNPs and 9 candidate genes significantly associated with drought tolerance were identified during the germination stage by using the GWAS method (Jia et al., 2024). Moreover, a number of 23 and 27 quantitative trait nucleotides linked with germination rate, whole seedling length, and root length were detected using MLM and mrMLM approaches, respectively (Aleem et al., 2024). However, due to drought tolerance being a complex quantitative trait with significant environmental interactions, the genetic basis at the germination stage in soybean remains unclear. In addition, some candidate genes have been shown to be functional under drought conditions. For example, *GmPLATZ17* (Zhao et al., 2022a), *GmbZIP15* (Zhang et al., 2020), and *GmLHY1a*/*GmLHY1b* (Wang et al., 2021) have been proven to be negative regulators of drought stress. While *GmNAC8* (Yang et al., 2020), *GmDREB1* (Chen et al., 2022), *GmNFYA13* (Ma et al., 2020a), *GmCOL1a* (Xu et al., 2023a), and *GmPrx16* (Zhang et al., 2024) have been shown to act as positive regulators in soybean drought stress. But these genes have been demonstrated to only function during the seedling or mature stages, leaving uncertainty about whether the identified genes exhibit similar adaptations during the germination stage.

With the development of next-generation sequencing technology, integration of RNA-seq and QTL mapping has been considered to be a reliable method to rapidly identify potential candidate genes. It has been applied to reveal QTL and candidate genes for nitrogen-deficiency tolerance in rice (Li et al., 2022b), identify SNPs and candidate genes associated with alkali stress tolerance at the germination stage in mung bean (Xu et al., 2023b), and uncover key drought-responding genes in barley seedlings (Xiong et al., 2023). Particularly, the weighted gene co-expression network analysis (WGCNA) has become an important tool in the identification of gene co-expression in relation to their functional associations and has been successfully used to analyze the transcriptome data of multiple samples in rice (Yu et al., 2020), maize (Liu et al., 2021), and rapeseed (Zhang et al., 2025) to identify key regulatory pathways and genes responsive to drought stress. However, no study has used this method to explain the gene networks and molecular regulatory mechanisms of soybean drought tolerance during the germination stage.

In the present study, the RTM-GWAS and RNA-seq methods were used to dissect the genetic basis of drought tolerance at the soybean seed germination stage. The main purposes of the present study were as follows: (1) to identify QTL associated with drought tolerance indices during the soybean germination stage, (2) to perform WGCNA to screen important gene co-expression modules and hub genes related to drought stress, and (3) to integrate of RTM-GWAS and RNA-seq analyses to screen the potential candidate genes controlling drought tolerance at the germination stage of soybean.

## 2 Materials and methods

### 2.1 Plant materials

A natural population of 207 soybean accessions collected from 11 provinces was used to conduct association mapping analysis. All of the soybeans were obtained from the Soybean Research Institute, College of Pharmacy and Life Science, Jiujiang University (Jiujiang, China).

### 2.2 Drought tolerance evaluation and statistical analysis

Twenty healthy and good-quality seeds were selected and sterilized with 1% NaClO_2_ for 30 s and then rinsed with distilled water (ddH_2_O) three times. Then the seeds were placed on two sheets of filter paper in sterilized 9 cm petri dishes. Drought stress was created by the addition of 15 ml polyethylene glycol 6000 (PEG-6000, 20% w/v) (Zhao et al., 2022b), whereas 15 ml ddH_2_O was employed in control conditions. The plates were incubated in a germination chamber at 25 ± 1°C, and the experiment was conducted in the dark for 6 days. Each processing was repeated three times. Subsequently, the treated seeds were rinsed by the corresponding solution (ddH_2_O or 20% (w/v) of PEG-6000), and 2 ml of the corresponding solution was added every day. The soybean seeds were considered to be germinated when the radicle length was beyond half of the longitudinal length of the seed. The count of germinated seeds was recorded every 24h for 6 days.

The germination traits were evaluated by the following formulas: Germination index (GI) = Σ (*G*t/*D*t), where *G*t is the accumulated number of germinated seeds on day *t*, and *Dt* indicates the germination days corresponding to *G*t in days; germination potential (GP) = (number of germinated seeds on the third day/total number of seeds) × 100%; germination rate (GR) = (number of germinated seeds on the sixth day/total number of seeds) × 100%. On the sixth day, for each cultivar, three normal and uniform growth seedlings were selected and used for phenotyping. The lengths of these radicles were measured by ruler as their seedling length (SL), and their fresh weights were measured by electronic balance and recorded as seedling fresh weight (SFW). The relative value of these traits was calculated by the formula: trait value under treatment/trait value of control × 100 (%), and then the relative germination index (RGI), relative germination potential (RGP), relative germination rate (RGR), relative seedling length (RSL), and relative seedling fresh weight (RSFW) were obtained.

The descriptive statistics, analysis of variance (ANOVA), and correlation analysis of the five germination-related traits were conducted using the programs MEANS, CORR, and PROC GLM by SAS 9.4 (SAS Institute, Cary, NC). The broad-sense heritability was estimated by the equation: $h^{2}=\sigma_{g}^{2}/\left(\sigma_{g}^{2}+\sigma_{e}^{2}/r\right)$ , where $\sigma_{g}^{2}$ (g=1, 2, 3,…207) is the genotypic variance, $\sigma_{e}^{2}$ represents random error variance, and *r* is the number of replicates (Nyquist and Baker, 1991).

### 2.3 Genotyping and population genetic analysis

The 207 soybean accessions were genotyped by ZDX1 (Sun et al., 2022), a high-throughput functional array, and a total of 123,540 SNPs were obtained (unpublished data). After filtering out SNPs with minor allele frequencies (MAF) ≤ 5%, a subset of 95,043 high-quality SNPs was used for further analyses.

The principal components analysis (PCA) and neighbor-joining tree were constructed using TASSEL 5.0 software (Bradbury et al., 2007). Linkage disequilibrium (LD) analysis was estimated using HAPLOVIEW 4.2 software. The 500-kb sliding window along each chromosome was used to calculate LD between all pairs of SNPs. The LD was estimated with the squared correlation coefficient *r* ^2^. The LD decay distance was defined as the chromosomal distance where the *r* ^2^ dropped to half of its maximum value (Barrett et al., 2005).

### 2.4 QTL mapping and candidate gene annotation

The RTM-GWAS procedure (https://github.com/njau-sri/rtm-gwas) was performed for QTL mapping (He et al., 2017). This association mapping was conducted in two stages. In the first stage, a single-locus model association analysis based on a simple linear model with a threshold of *P* = 0.05 was performed to preliminarily screen markers. In the second stage, the significance level of *P* = 0.05 under the multi-locus model was used for the preselected markers to identify genome-wide QTLs through stepwise regression. Manhattan and quantile–quantile plots were generated using the R software. The nomenclature (McCouch et al., 1997) with modifications was used to name the QTL in this study. Such as *qRGI-3-1*, *q* represents the QTL; *RGI* represents the relative germination index, *-3* represents chromosome 3; *-1* represents the first QTL on that chromosome. Genes located within ± 200 kb (the average LD decay distance across all 20 chromosomes) of the QTLs were identified as candidate genes. The Phytozome database (http://www.phytozome.org/) and the SoyBase database (http://www.soybase.org/) were used to annotate the candidate genes.

### 2.5 RNA−seq and data analysis

Two accessions, JJS98 (high drought-tolerant, DT) and JJS294 (high drought-sensitive, DS), were selected from the association panel for transcriptomic analysis. Briefly, the seeds of JJS98 and JJS294 were germinated under normal conditions, and after five days the germinating seeds were transferred into the ddH_2_O or 20% (w/v) of PEG-6000. For each accession, the root tips were respectively collected at 0h, 12h, and 24h after drought treatment, with three biological repetitions. The total RNA was isolated using a Plant RNA Extract Kit (TianGen, Beijing, China) according to the manufacturer’s instructions. RNA integrity was assessed using the RNA Nano 6000 Assay Kit of the Bioanalyzer 2100 system (Agilent Technologies, CA, United States) (**Supplementary Table S1**). In total, 18 RNA samples were submitted to Biomarker Technologies Co., Ltd. (Beijing, China) for sequencing using the Illumina HiSeq 4000 sequencing platform.

The raw paired-end reads were first filtered with Fastp software to obtain high-quality clean data and then aligned to the soybean reference genome Williams 82 (*Glycine max* v2.1 genome) using Hisat2 v2.0.5 software. The FPKM (fragments per kilobase of transcript per million fragments mapped) method was utilized to normalize and estimate gene expression values. DEGs were identified using the false discovery rate (FDR) < 0.01 and |log_2_^FoldChang^| ≥2. Gene Ontology (GO) and Kyoto Encyclopedia of Genes and Genomes (KEGG) enrichment analyses were performed using BMKCloud (www.biocloud.net).

### 2.6 Weighted gene co-expression network analysis

The co-expression network was constructed in R using the WGCNA (v1.47) package. For high reliability of the results, the FPKM greater than 1 of all DEGs was selected for WGCNA analysis. According to the correlation of gene expression, the gene clustering tree was constructed, and then the genes with similar expression patterns were classified into the same module. The modules were merged with the threshold value of module eigenvalue similarity > 0.25, and the minimum gene number of modules was set as 30 (Wang et al., 2024). The value of the soft threshold (β) was selected as 13, the hierarchical clustering tree was a dynamic hybrid tree cut algorithm, and the weighted network was unsigned. Modules that are significantly correlated with specific samples are identified according to the heatmap of the sample expression patterns, and the DEGs in these modules were used for KEGG enrichment analysis on the BMKCloud platform. In each module, the genes with eigengene-based connectivity value (|KME|) > 0.98 were regarded as hub genes.

### 2.7 Validation of the DEGs by qRT−PCR

Five hub genes were selected for qRT-PCR validation. Total RNA was extracted from the same samples that were used for sequencing. First-strand cDNA was synthesized using a HiScript IV RT SuperMix for qPCR (+gDNA wiper) (Vazyme Biotech Co., Ltd, Nanjing, China). The qRT-PCR experiment was performed on a CFX Connect (TM) Real-Time PCR Detection System (BIO-RAD, Singapore) using SupRealQ Purple Universal SYBR qPCR Master Mix (U+) (Vazyme Biotech Co., Ltd, Nanjing, China) according to the instructions. Soybean gene *GmActin11* (*Glyma.18G290800*) was the internal control gene, and fold change was calculated using the 2^−ΔΔCT^ method (Livak and Schmittgen, 2001). The detail of primers was shown in **Supplementary Table S2**.

## 3 Results

### 3.1 Phenotypic variation and correlation analysis

Five germination-related traits, namely GI, GP, GR, SL, and SFW, were measured for germinating seeds of the 207 soybean accessions under ddH_2_O (CK) or 20% (w/v) of PEG-6000 (PEG) (**Table 1**). The mean values of GI, GP, GR, SL, and SFW for the seeds treated with CK were 17.72, 0.59, 0.67, 7.59, and 2.02, while the means for the PEG were 6.21, 0.12, 0.56, 2.40, and 1.41, respectively. Obviously, all of these traits were substantially higher under the control group than under drought-stressed conditions, indicating that after 20% PEG-6000 drought treatment, the germination of soybean germplasms was greatly inhibited. Moreover, a descriptive statistical analysis was also conducted on RGI, RGP, RGR, RSL, and RSFW of 207 soybean accessions. The average values of RGI, RGP, RGR, RSL, and RSFW were 0.36, 0.22, 0.83, 0.37, and 0.71, respectively. The coefficients of variation (CVs) of the RGI, RSL, RGR, and RSFW were 89.69%, 63.53%, 59.60%, and 26.04%, respectively. The CVs of RGP were 168.91%, surpassing the 100% threshold. These indicated that the population used in this study exhibits rich variation under drought conditions. Besides, the heritability of all five germination-related traits was high and ranged from 65.25% to 97.38% (**Table 1**), indicating that most of the phenotypic variance of seed-droughting tolerance was mainly controlled by genetic variation.

**Table 1:** Descriptive statistics of five germination-related traits under ddH_2_O (CK) or 20% (w/v) of PEG-6000 (PEG) conditions for germinating seeds in the 207 soybean accessions.

Trait	Min.	Max.	Range	Mean	SD	CV (%)	Skewness	Kurtosis	*h* ^2^ (%)
GI_CK	0.00	48.00	48.00	17.72	9.38	52.93	0.15	−0.34	96.51
GP_CK	0.00	1.00	1.00	0.59	0.29	49.05	−0.39	−0.99	96.12
GR_CK	0.00	1.00	1.00	0.67	0.27	40.77	−0.68	−0.56	95.22
SL_CK	0.80	17.70	16.90	7.59	3.67	48.37	0.26	−0.93	94.58
SFW_CK	0.50	3.57	3.07	2.02	0.45	22.21	0.12	0.15	90.18
GI_PEG	0.00	19.93	19.93	6.21	4.76	76.65	0.71	−0.17	97.38
GP_PEG	0.00	0.85	0.85	0.12	0.16	138.18	1.98	4.14	93.92
GR_PEG	0.00	1.00	1.00	0.56	0.32	57.01	−0.32	−1.18	96.35
SL_PEG	0.30	6.20	5.90	2.40	1.06	43.98	0.33	−0.33	91.66
SFW_PEG	0.56	4.23	3.67	1.41	0.33	23.21	1.78	11.15	95.15
RGI	0.00	3.40	3.40	0.36	0.33	89.69	3.79	25.34	65.98
RGP	0.00	5.00	5.00	0.22	0.36	168.91	6.11	63.69	65.25
RGR	0.00	4.50	4.50	0.83	0.50	59.60	1.85	10.64	71.07
RSL	0.03	1.94	1.91	0.37	0.23	63.53	2.39	9.23	76.47
RSFW	0.38	2.62	2.24	0.71	0.18	26.04	3.11	24.24	78.87

CK and PEG represent under the control group and under drought-stressed conditions, respectively. GI, germination index; GP, germination potential; GR, germination rate; SL, whole seedling length; SFW, seedling fresh weight. RGI, relative germination index; RGP, relative germination potential; RGR, relative germination rate; RSL, relative seedling length; RSFW, relative seedling fresh weight. SD, standard deviation; CV, coefficient of variation; *h* ^2^, heritability.

An ANOVA indicated that there were significant (*P <* 0.001) differences in the five germination-related traits among genotypes, treatments, and genotype-by-treatment interactions. However, there was no significant variation in the five germination-related traits among treatments (replications) (**Table 2**), indicating experimental data is relatively reliable.

**Table 2:** Analysis of variance of the five germination-related traits under ddH_2_O (CK) or 20% (w/v) of PEG-6000 (PEG) conditions for germinating seeds in the 207 soybean accessions.

Trait	Variation	DF	SS	MS	*F*-value	*P-*value
GI	Genotype	206	47211.29	229.18	44.01	<.0001
	Treatment	1	36889.31	36889.31	7083.82	<.0001
	Treatment (Replication)	4	8.25	2.06	0.40	0.8117
	Genotype*Treatment	205	13886.70	67.74	13.01	<.0001
	Error	760	3957.73	5.21		
GP	Genotype	206	39.81	0.19	28.97	<.0001
	Treatment	1	62.14	62.14	9315.73	<.0001
	Treatment (Replication)	4	0.01	0.00	0.29	0.882
	Genotype*Treatment	205	20.15	0.10	14.74	<.0001
	Error	772	5.15	0.01		
GR	Genotype	206	85.27	0.41	40.63	<.0001
	Treatment	1	3.69	3.69	362.31	<.0001
	Treatment (Replication)	4	0.03	0.01	0.81	0.5199
	Genotype*Treatment	205	11.85	0.06	5.68	<.0001
	Error	774	7.88	0.01		
SL	Genotype	205	4764.76	23.24	20.68	<.0001
	Treatment	1	7066.49	7066.49	6287.13	<.0001
	Treatment (Replication)	4	1.58	0.39	0.35	0.8435
	Genotype*Treatment	196	2645.69	13.50	12.01	<.0001
	Error	718	807.00	1.12		
SFW	Genotype	205	113.71	0.55	17.32	<.0001
	Treatment	1	93.75	93.75	2927.56	<.0001
	Treatment (Replication)	4	0.13	0.03	1.01	0.4019
	Genotype*Treatment	196	33.08	0.17	5.27	<.0001
	Error	709	22.70	0.03		

GI, germination index; GP, germination potential; GR, germination rate; SL, whole seedling length; SFW, seedling fresh weight; DF, degree of freedom; SS, sum of squares; MS, mean square.

To explore the correlation among RGI, RGP, RGR, RSL, and RSFW, Pearson’s correlation analysis was conducted based on the means for the 207 accessions (**Table 3**). The result showed that there were significant positive correlations for all the five germination-related traits. In particular, RGI exhibited a significant positive correlation with RGP, RGR, and RSL (*P <* 0.001), with large correlation coefficients of 0.86, 0.82, and 0.62, respectively (**Table 3**). These results demonstrated that there is a certain correlation between different drought-tolerant traits.

**Table 3:** Pearson correlation coefficients between five germination-related traits in the 207 soybean accessions.

Trait	RGI	RGP	RGR	RSL	RSFW
RGI	1.00				
RGP	0.86**	1.00			
RGR	0.82**	0.59**	1.00		
RSL	0.62**	0.53**	0.46**	1.00	
RSFW	0.17*	0.13	0.03	0.41**	1.00

GI, germination index; GP, germination potential; GR, germination rate; SL, whole seedling length; SFW, seedling fresh weight. * and ** represent significant at the levels of *P* < 0. 05 and *P* < 0. 001, respectively.

### 3.2 Population structure and linkage disequilibrium analysis

The neighbor-joining tree and PCA analyses were used to determine the population structure of 207 soybean accessions. Based on the neighbor-joining tree analysis, these 207 accessions were clearly grouped into three groups (**Supplementary Figure S1A**). For the three groups, the group 3 accessions were primarily from Southern China, the Huanghuai region, and local collections, while the Groups 2 and 1 accessions mainly originated from Hebei, Fujian, and Guangxi provinces. Furthermore, the result of the PCA analysis was consistent with the neighbor-joining tree analysis (**Supplementary Figure S1B**).

In the present study, the whole-genome LD decay rate of the population was estimated. Calculation results showed that the maximum *r* ^2^ value was 0.71. Then, when the *r* ^2^ value dropped to half of its maximum value, the LD decay distance reached approximately 200 kb (**Supplementary Figure S2**).

### 3.3 Identification of significant loci for the drought tolerance using RTM-GWAS

**Figure 1:**
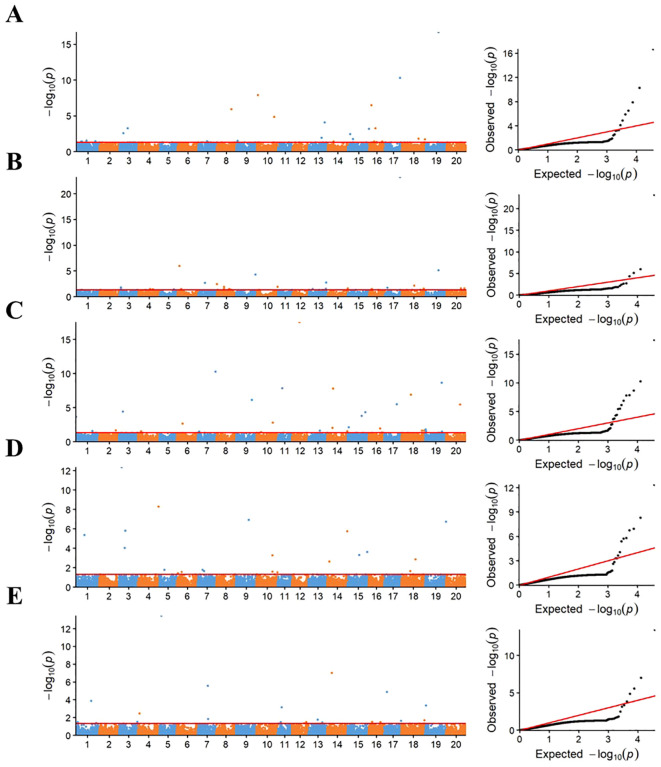
Manhattan and quantile-quantile plots of RTM-GWAS for five germination traits. **(A–E)** represent the Manhattan and quantile-quantile plots of RTM-GWAS for Relative germination index (RGI), Relative germination potential (RGP), Relative germination rate (RGR), Relative whole seedling Length (RWSL), and Relative seedling fresh weight (RSFW) in turn; the horizontal red line represents the genome-wide significance threshold of 0.05, where the (−log_10_ *P*) values was 1.10.

The RTM-GWAS method was used to perform association analysis on five germination-related traits. A total of 58 QTL were detected with a significance level of *P* < 0.05 (**Figure 1** and **Supplementary Table S3**). The -log *P* values of these QTLs ranged from 2.03 to 23.22 and explained 1.11% to 40.63% of the genetic contribution (*R* ^2^).

Among these QTLs, there were 12 QTLs for RGI, and the phenotypic variation of these loci was 1.47% to 24.12%, which together explained a total of 84.36% of genetic contribution. Eight QTLs were identified for RGP, and the phenotypic variance varied from 2.20% to 44.36%, collectively accounting for 71.38% of the genetic contribution. There were 17 QTLs for RGR, and the phenotypic variation was 1.11%–19.28%, explaining a total of 88.35% of genetic contribution. Thirteen QTLs were identified for RSL, and the phenotypic variance varied from 2.31% to 12.58%, collectively accounting for 84.39% of the genetic contribution. For 8 RSFW QTLs, the range of phenotypic variation was 2.99% to 40.63% and explained a total of 78.22% of genetic contribution (**Supplementary Table S3**). More importantly, the two pairs of QTLs, *qRGI-17–1* and *qRGP-17-1*, and *qRGI-19–1* and *qRGP-19-1*, were located on the same genomic region. *qRGI-17–1* and *qRGP-17–1* were mapped to the same SNP of Gm17_BLOCK_37041250_37044388, accounting for 24.12% and 44.36% of the phenotypic variation, respectively. *qRGI-19–1* and *qRGP-19–1* were co-associated with the SNP marker Gm19_BLOCK_30608933_30612180, explaining 15.95% and 5.77% phenotypic variation, respectively. Out of these QTLs, 16 QTLs were co-localized with the previously reported soybean drought tolerance QTL, and the others were identified in this study (**Supplementary Table S3**).

In addition, loci with a higher phenotype variation (*R* ^2^>10%) were considered as large-contribution QTLs in this study, including *qRGI-16-1*, *qRGI-17-1*, *qRGI-19-1*, *qRGP-17-1*, *qRGR-7-1*, *qRGR-12-1*, *qRGR-15-1*, *qRSL-3-1*, *qRSFW-5-1*, and *qRSFW-14-1*. These large-contribution QTLs were used for the following candidate gene mining.

### 3.4 Transcriptome analysis for identifying DEGs

Via transcriptome sequencing for the two accessions with contrasting drought tolerance, a total of 137.74 Gb of clean data were obtained from 18 libraries. In different samples, the Q30 percentage was over 94.42%, and more than 90.56% of clean reads were mapped to the soybean genome, while the percentage of unique mapped reads ranged from 88.61 to 93.63% (**Supplementary Table S4**), indicating that the RNA-seq libraries are of high quality. Based on three biological replicates at different time points after drought treatment, the PCA was conducted on all expressed genes in 18 samples. The first two principal components, PC1 and PC2, accounted for 24.35% and 15.72%, respectively (**Supplementary Figure S3A**). These results indicated that there were different gene expression patterns among the soybean roots under drought stress at different time points (**Supplementary Figure S3B**).

**Figure 2:**
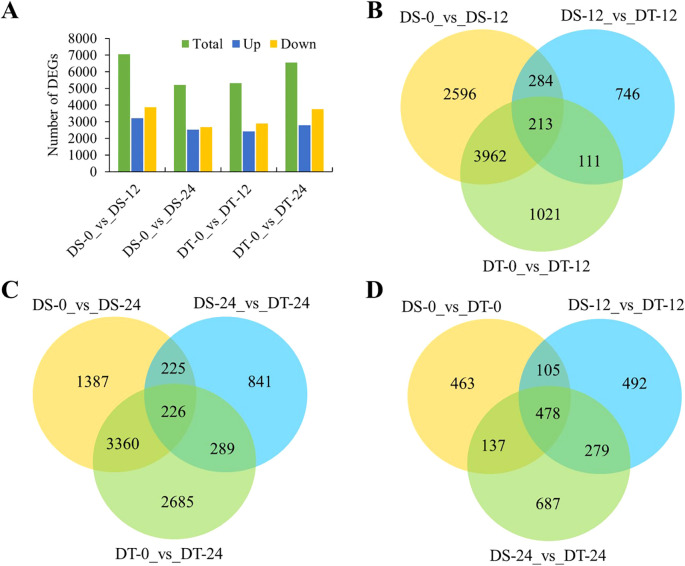
Time-dependent comparisons of DEGs. **(A)** Number of drought-responsive genes identified at two time points. **(B, C)**, Venn diagrams showing the extent of overlap between DEGs from DS and DT after 12h **(B)**, or 24h **(C)** of drought treatment. **(D)**, Venn diagram showing DEGs across the three time points.

Subsequently, the analysis of differentially expressed genes (DEGs) was performed in two aspects. On the one hand, drought-responsive genes were determined by comparing drought-treated samples with controls for two accessions at two time points. Relative to the gene expression levels at 0h, 7,055 (3,196 upregulated and 3,859 downregulated) and 5,198 (2,509 upregulated and 2,689 downregulated) DEGs were detected in the DS accession at 12h and 24h after drought treatment, respectively. While 5,307 (2,414 upregulated and 2,893 downregulated) and 6,560 (2,792 upregulated and 3,768 downregulated) DEGs were detected in the DT accession at 12h and 24h after drought treatment, respectively (**Figure 2A**). There were 2,041 DEGs continuously responding to drought stress both in DS and DT accessions (**Supplementary Figure S4**). On the other hand, DEGs between the two accessions at two time points were identified. There were 608 DEGs after 12h of drought treatments and 740 DEGs after 24h between the two accessions, which did not include DEGs that are not drought-responsive (**Figures 2B, C**). The number of DEGs identified after 24h of drought treatments more than 12h suggested that more gene networks are triggered by drought stress in the two accessions with time increasing. Of the 608 DEGs after 12h, 213 (35.0%) were shared by DS and DT, while the remaining 284 (46.7%) and 111 (18.3%) were DS- and DT-specific DEGs, respectively (**Figure 2B**). By contrast, the relative proportion of DEGs identified after 24h of drought treatment was equivalent to after 12h, as 226 of 740 DEGs (30.5%) were shared by DS and DT, and 225 (30.4%) and 289 (39.1%) uniquely belonged to DS and DT accessions, respectively. These results indicated that accession-specific responses to drought stress occurred (**Figure 2C**). In addition, 1,183, 1,354, and 1,581 DEGs between DS and DT after 0h, 12h, and 24h of drought treatment were separately obtained, and 478 genes showed significant expression differences between DS and DT at all time points (**Figure 2D**). Two DEGs were commonly detected in all pairwise comparison groups (**Supplementary Figure S5**).

### 3.5 Functional enrichment analysis of DEGs by GO and KEGG

**Figure 3:**
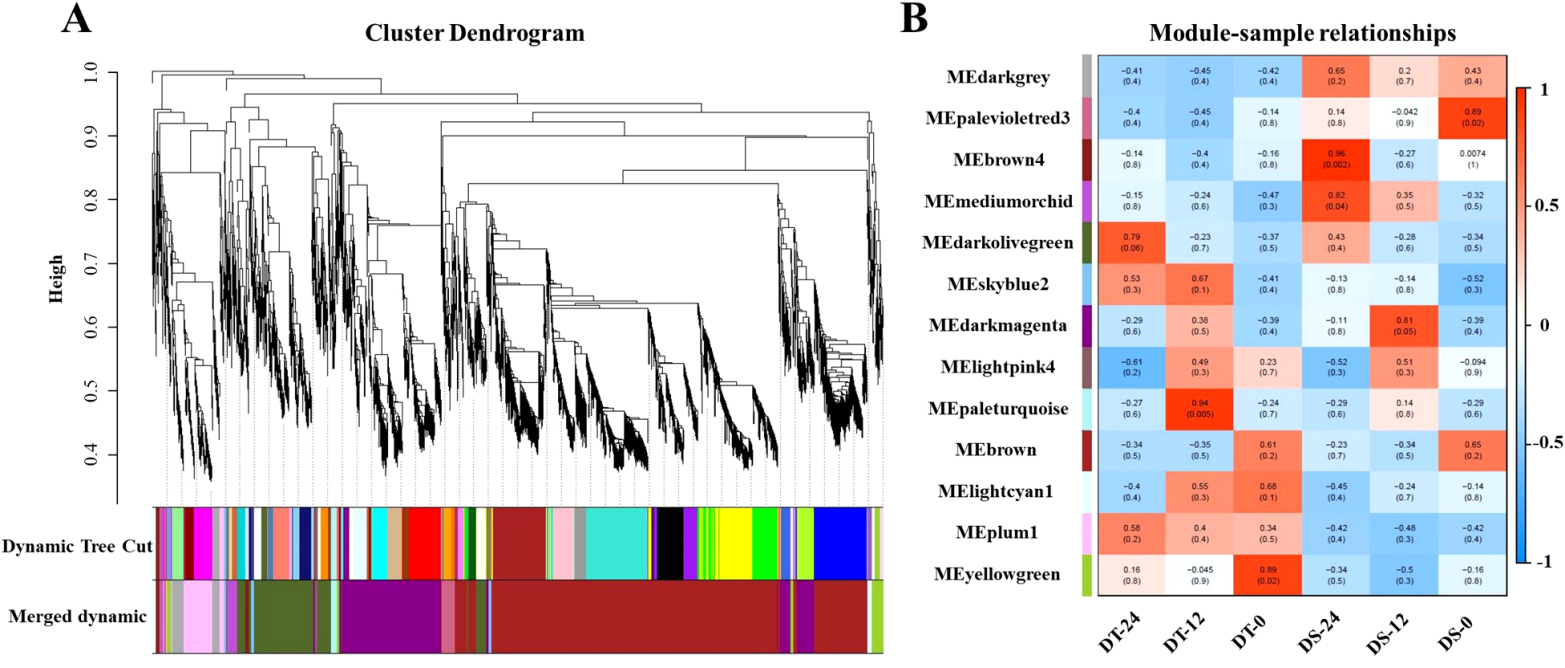
KEGG enrichment analysis for DEGs between DS and DT at two time points. **(A, B)**, the top 20 KEGG pathway with the highest significance after 12h and after 24h of drought treatment, respectively.

To know the functional information of the DEGs between DS and DT at two time points, the GO enrichment and KEGG analyses were performed. GO enrichment analysis indicated that 1,354 DEGs identified after 12h of drought treatment were mainly concentrated in hydrogen peroxide catabolic process (GO:0042744), response to oxidative stress (GO:0006979), root morphogenesis (GO:0010015), and signal transduction (GO:0007165) (**Supplementary Figure S6A**). Interestingly, the GO terms, such as hydrogen peroxide catabolic process (GO:0042744) and response to oxidative stress (GO:0006979), were also significantly enriched after 24h of drought treatment (**Supplementary Figure S6B**). While the GO term of glutathione metabolic process (GO:0006749) was only significantly enriched after 24h (**Supplementary Figure S6B**). KEGG analysis showed that three identical pathways were significantly enriched after 12h and 24h of drought treatment, including phenylpropanoid biosynthesis, starch and sucrose metabolism, and fatty acid degradation (**Figures 3A, B**). In the phenylpropanoid biosynthesis pathway, 14 of 32 DEGs after 12h of drought treatments and 19 of 42 DEGs after 24h were annotated as peroxidase activity, which corresponded to the results of GO enrichment analysis (**Supplementary Table S5**). These observations indicated that the enrichment of antioxidant and secondary metabolite pathways was related to the ability of soybean drought tolerance at the germination stage.

Besides, 2,041 drought-responsive DEGs shared by DS and DT at two time points were also analyzed. GO analysis revealed a stark enrichment of drought-responsive DEGs in several functional categories, namely hydrogen peroxide catabolic process (GO:0042744), response to oxidative stress (GO:0006979), and UDP-glycosyltransferase activity (GO:0008194) (**Supplementary Figure S7**). The KEGG annotations indicated that the pathways enriched with these drought-responsive DEGs were closely related to phenylpropanoid biosynthesis, glutathione metabolism, isoflavonoid biosynthesis, and the MAPK signaling pathway (**Supplementary Figure S8**).

### 3.6 Co-expression network analysis of DEGs

**Figure 4:**
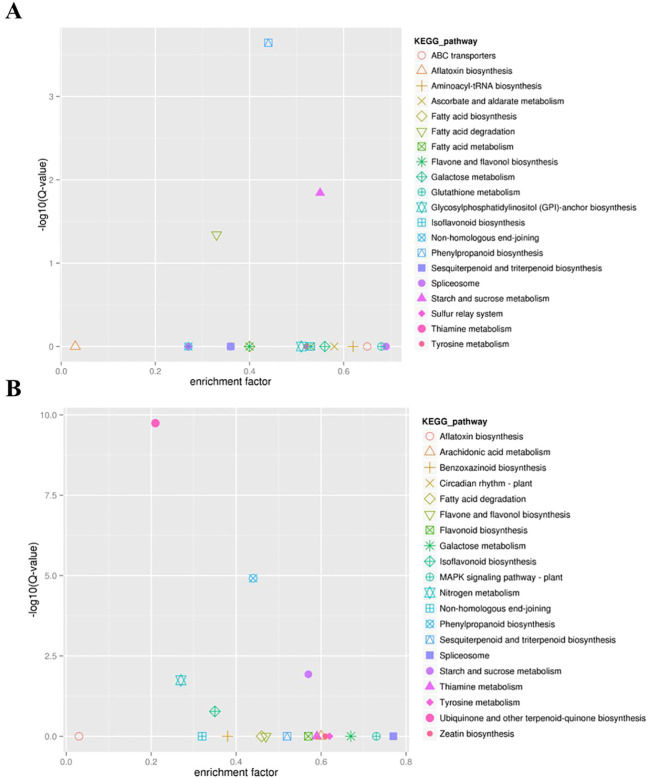
WGCNA analysis of all DEGs identified in this study. **(A)** Gene cluster dendrograms and module detecting. **(B)** Module-sample relationships based on Pearson correlation coefficients. Each row corresponds to a module indicated by different colors. Each column corresponds to a sample from different times of DT or DS. The right panel shows positive (red, 1) and negative (blue, −1) correlations.

**Figure 5:**
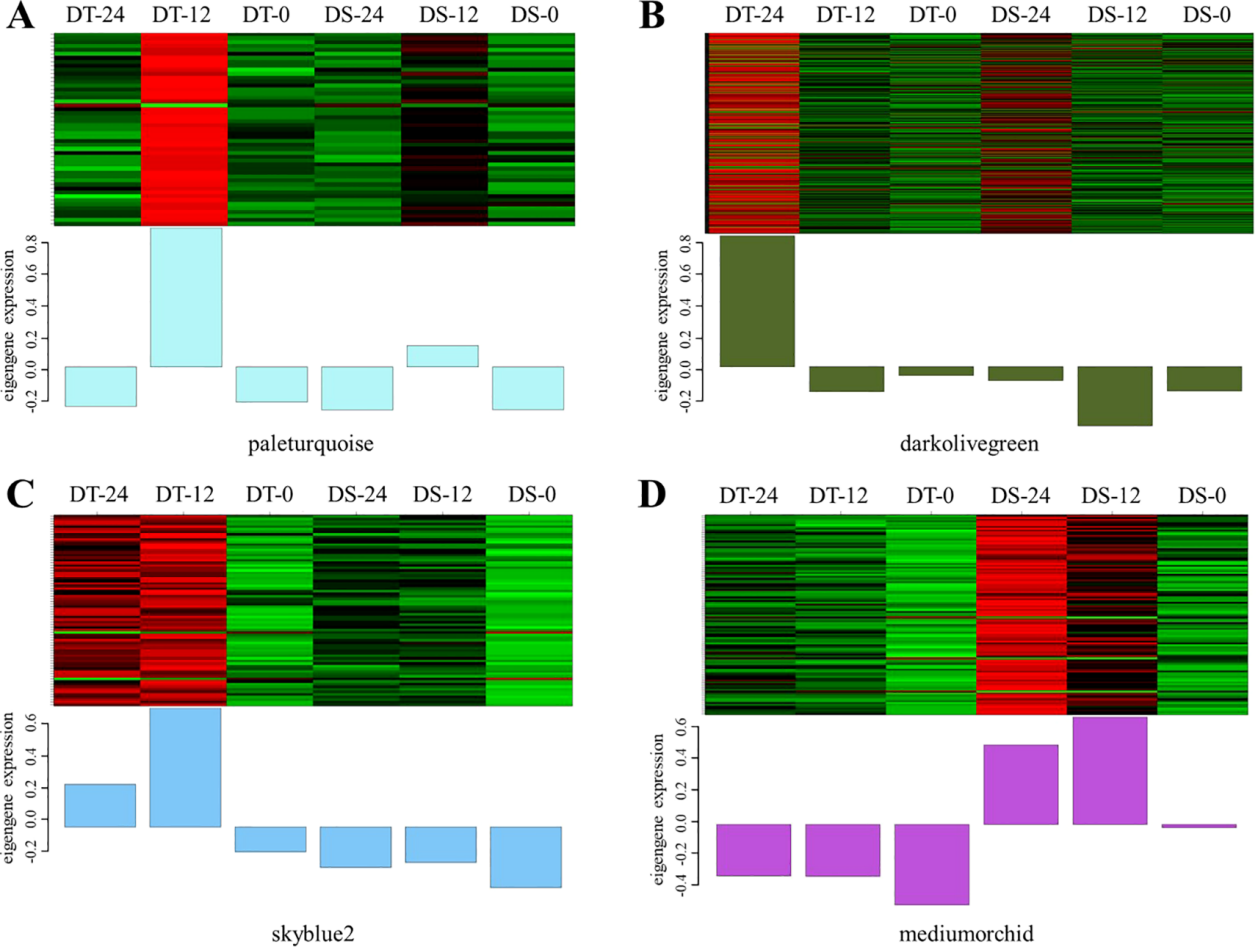
DEGs expression heatmap of four co-expression modules in different samples. **(A–D)** represent the expression heatmap of DEGs in the paleturquoise, darkolivegreen, skyblue2, and mediumorchid modules in different samples, respectively.

To further identify the hub genes or modules that are associated with the drought stress response in soybean roots, all DEGs between two accessions and between treatments at different times were applied for WGCNA analysis. Based on the co-expression patterns of these DEGs, the gene cluster dendrogram was constructed, and 13 distinct modules (labeled with different colors) were identified (**Figure 4A**). Next, the expression patterns of each module in different samples were analyzed based on the module eigenvalues (**Figure 4B**). Compared to other samples, the DT-12 and DT-24 samples were most significantly correlated with the pale turquoise and dark olive green modules, respectively. While both of these two samples were significantly correlated with the skyblue2 module. In contrast, the medium orchid module showed positive correlation with both DS-12 and DS-24 samples (**Figure 4B**). Therefore, the above-mentioned four co-expression modules were considered as key modules for further analysis. Analysis of the expression mode of DEGs showed that there was significant specificity in the expression of DEGs in each key module. For example, the pale turquoise and dark olivegreen modules respectively represent DEGs that showed high expression specifically at 12h and 24h after drought treatment in DT accession (**Figures 5A, B**). Additionally, DEGs in the skyblue2 and medium orchid modules were continuously highly expressed after drought treatment in DT and DS accessions, respectively (**Figures 5C, D**).

Furthermore, KEGG pathway enrichment analysis showed that the highly enriched pathways in the paleturquoise module included phenylpropanoid biosynthesis, starch and sucrose metabolism, and peroxisome (**Supplementary Figure S10A**). The highly enriched pathways in the dark olive green module included phenylpropanoid biosynthesis, fatty acid degradation, and the MAPK signaling pathway (**Supplementary Figure S10B**). Similarly, the skyblue2 module showed enrichment of KEGG pathways related to glutathione metabolism, phenylpropanoid biosynthesis, and plant hormone signal transduction (**Supplementary Figure S10C**). The medium orchid module is involved in glutathione metabolism, isoflavonoid biosynthesis, and zeatin biosynthesis pathways (**Supplementary Figure S10D**).

Additionally, a total of 40 hub genes were discovered in the four modules according to the kME value (|KME| > 0.98) and gene annotation, of which 28, 3, 4, and 5 existed in the dark olive green, medium orchid, pale turquoise, and skyblue2 modules, respectively (**Supplementary Table S6**). Most strikingly, in the dark olive green module, *Glyma.13G229300* encodes abscisic acid receptor PYL11-related protein, and *Glyma.08G188300* encodes SNF1-related protein kinases 2 (SnRK2s), which are two key elements of ABA signaling that play an essential role in the abiotic stress response. Four genes (*Glyma.05G226700*, *Glyma.16G181200*, *Glyma.03G066700*, and *Glyma.03G066600*) were related to the oxidation-reduction process that is essential for basic life functions, including photosynthesis and respiration. Two transcription factors, *BHLH57* and *NF-YA8*, respectively encoded by *Glyma.12G178500* and *Glyma.17G051400*, were identified as hub genes. Furthermore, *Glyma.04G231400*, encoding an HD-ZIP family transcription factor; *Glyma.09G052800*, encoding an ethylene-responsive transcription factor; *Glyma.12G091200* encoding an NAC transcription factor; and *Glyma.13G124900*, encoding a GRAS domain transcription factor, were identified as hub genes in the medium orchid, pale turquoise, and skyblue2 modules, respectively.

### 3.7 Candidate gene screening by integration of RTM-GWAS and RNA-seq

Among the 10 large-contribution QTLs, a total of 294 genes were retrieved within ± 200 kb (average LD decay distance across all 20 chromosomes) of these QTLs, according to the soybean genome Williams 82 (*Glycine max* v2.1 genome) (**Supplementary Table S7**). Among the 294 genes detected by RTM-GWAS, three genes are differentially expressed between DS and DT accessions, and 19 genes belong to drought-responsive DEGs in both accessions, according to the transcriptome data (**Supplementary Table S8**). Herein, these 22 genes were identified as putative candidate genes.

**Figure 6:**
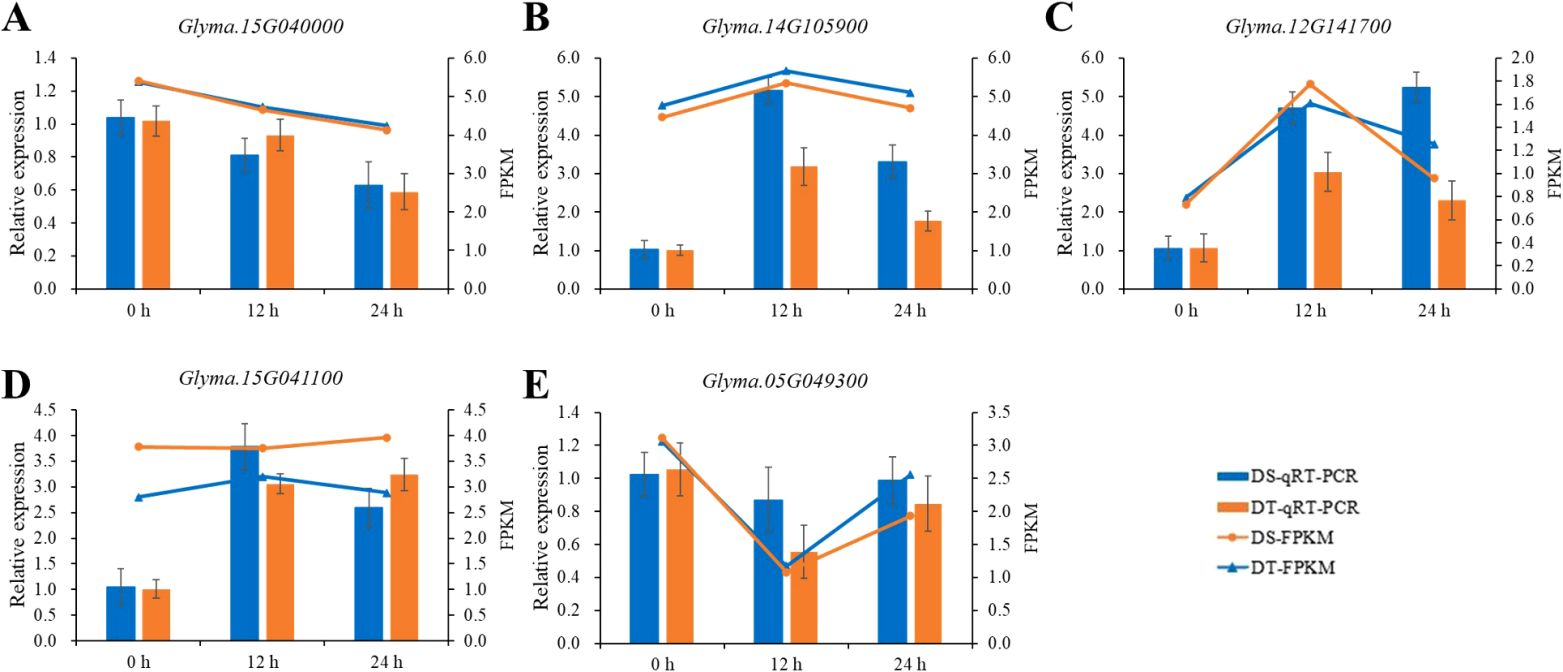
The expression analysis of five priority candidate genes. The histograms represent relative expression levels of *Glyma.15G040000* **(A)**, *Glyma.14G105900* **(B)**, *Glyma.12G141700* **(C)**, *Glyma.15G041100* **(D)**, and *Glyma.05G049300* **(E)** as determined by qRT-PCR and the line charts represent FPKM values from the RNA-seq data. The FPKM value was normalized by log_2_^(FPKM+1)^.

According to the functional annotations, the five candidate genes, *Glyma.12G141700*, *Glyma.15G040000*, *Glyma.05G049300*, *Glyma.14G105900*, and *Glyma.15G041100*, were further considered as priority candidate genes. Among them, three transcription factors, *Glyma.12G141700*, *Glyma.05G049300*, and *Glyma.15G041100*, belong to the bHLH gene family, GRAS gene family, and MYB gene family, respectively; *Glyma.15G040000* and *Glyma.14G105900* encode a cytosolic isoform of UDP-glucuronic acid decarboxylase and an E3 ligase, respectively. Among these genes, the transcriptome data uncovered that the *Glyma.15G040000* and *Glyma.05G049300* genes were downregulated both in DT and DS after 12 and 24h of drought treatment, whereas *Glyma.12G141700* and *Glyma.14G105900* were upregulated. Besides, *Glyma.15G041100* was downregulated at 12h and upregulated at 24h after drought treatment in DT, while it was upregulated both at 12 and 24h after drought treatment in DS (**Figure 6**).

### 3.8 Validation of the priority candidate genes by RT-qPCR

To confirm the accuracy of the RNA sequencing results, five priority candidate genes were selected for qRT-PCR analysis. The qRT-PCR results of five genes were consistent with the expression pattern of RNA-seq data (**Figure 6**). Genes significantly up-regulated in RNA-Seq data also exhibited an upregulation in qRT-PCR, and vice versa, suggesting the reliability and accuracy of our RNA-Seq data.

## 4 Discussion

Drought stress is one of the most significant environmental conditions adversely affecting world agricultural production (Cattivelli et al., 2008; Dai, 2013). Of the legumes, soybean is the most sensitive to water (Condon et al., 2004). Seed germination is the onset of plant growth and development (Waterworth et al., 2015), and drought stress during this stage can reduce the total number of seedlings by 20%, and in severe cases, it can even decrease yield by more than 50% (Devi et al., 2014; Zhao et al., 2017). It is crucial to understand drought tolerance mechanisms during the soybean germination stage for developing new drought-resistant varieties of soybean by new genetic engineering techniques.

Many indicators were employed to evaluate drought resistance during the soybean germination stage. For instance, DT-RW, DT-RL, and DT-GR were selected as drought-tolerance indices to examine the phenotypes of a panel of 259 released Chinese soybean cultivars (Liu et al., 2020). Five drought tolerance indices: RGR, RGE, GDRI, GSI, and MFV were used to examine the drought-tolerance phenotypes of 410 soybean accessions in the germination stage (Zhao et al., 2022b). Recently, the researchers used GR, GE, GI, RGR, RGE, RGI, and ASFV as the drought tolerance indicators to evaluate a natural population of 264 Chinese soybean accessions with 2,597,425 SNPs (Jia et al., 2024). The choice of drought resistance indicators directly affects the reliability and accuracy of the experimental results. Taking into account the above results, our study adopts RGI, RGP, RGR, RSL, and RSFW as evaluation indicators for soybean drought resistance during the germination stage. Herein, the broad-sense heritability of those five traits displayed high (65.25%–78.87%), suggesting the variation was largely due to genetic effects and demonstrating the possibility of selecting for drought tolerance during breeding of climate-resilient soybean cultivars. Besides, we also found that RGI were all positively correlated with RGP, RGR, RSL, and RSFW (*P <* 0.05), which reflected the inherent relations among the original drought-tolerant indicators that can be used for drought-tolerant identification.

Drought tolerance is a complex quantitative trait, and many QTLs distributed on most chromosomes have been detected at the soybean germination stage in several studies. For example, 15 SNPs associated with the drought-tolerance index were identified (Liu et al., 2020). 26 SNP loci related to drought tolerance during the germination stage were detected by GWAS (Zhao et al., 2022b). In this study, 58 QTLs were detected to be associated with five drought tolerance indices using the RTM-GWAS method. Among them, 14 QTLs have been detected in previous studies using various drought tolerance-related traits at the germination stage (**Supplementary Table S3**). Of these, *qRGI-17-1*, explaining 24.12% phenotypic variation, overlaps with the ss249472124 marker associated with both DT-GR and DT-RL (Liu et al., 2020). *qRGR-20–1* is located near the previously reported SNP S20_32847223 associated with drought-tolerance index RGE (Jia et al., 2024).

Furthermore, we compared the results of this study with previously studied QTLs of drought tolerance at other developmental stages. There are 16 QTLs that have been detected in previous studies at the seedling or maturation stages (**Supplementary Table S3**). For instance, *qRGR-11–1* overlaps with the previously mapped QTL of *mqCanopy wilt-020* (Hwang et al., 2016) and *Canopy wilt 4-4*, *Canopy wilt 4-7*, and *Canopy wilt 5-1* (Hwang et al., 2015). *qRGR-11–1* was also co-identified in other QTLs of *qWS-11–2* and *qDTW-11-2*, and *Glyma.11g143500* (*GmUAA6*, encoding a UDP-N-acetylglucosamine (UAA) transporter) within this drought tolerance QTL was found as a novel candidate gene associated with drought tolerance (Jiang et al., 2024). Additionally, we defined the major QTLs with *R* ^2^ > 10%, and there are a total of 10 QTLs that meet the criteria, including *qRGI-16-1*, *qRGI-17-1*, *qRGI-19-1*, *qRGP-17-1*, *qRGR-7-1*, *qRGR-12-1*, *qRGR-15-1*, *qRSL-3-1*, *qRSFW-5-1*, and *qRSFW-14-1*. Of those, *qRSFW-5–1* had the second largest phenotypic variation of 40.63% in this study, which overlapped with the *Canopy wilt 2-3* (Abdel-Haleem et al., 2012), *Canopy wilt 3-1* (Hwang et al., 2015), and *Drought tolerance 6-2* (Carpentieri-Pipolo et al., 2012). The overlapping of QTL identified in this study with the published QTL for soybean drought tolerance suggests the accuracy of these QTL. Furthermore, *Glyma.16g211700*, a candidate gene within the *qRGI-16–1* locus identified in our study, was validated as a key drought-tolerant gene in soybean through an integrative data-driven feature engineering pipeline (Kao et al., 2025). Interestingly, three drought-tolerant QTLs identified at the germination stage could also be identified at the seedling stage, which suggested that some genes may simultaneously control the drought tolerance of soybean at the seedling and germination stages. In fact, several common QTLs have been identified for the rice salt tolerance at the germination and seedling stages (Nakhla et al., 2021). So, we considered that the remaining 31 QTLs are novel drought tolerance loci.

From our transcriptome data, it can be seen that thousands of genes are involved in the drought tolerance response at the germination stage in soybean. To reduce the number of potential candidate genes, the WGCNA analysis was performed. Based on the module eigenvalues, dark olive green, medium orchid, pale turquoise, and skyblue2 modules were selected from 13 distinct modules, and then 40 key candidate genes were identified from these four key modules. The KEGG analysis showed that the phenylpropanoid biosynthesis pathway was highly enriched in three out of these four key modules. Moreover, the KEGG pathways of the DEGs between DS and DT at two time points and drought-responsive DEGs shared by DS and DT also included the phenylpropanoid biosynthesis pathway. These findings suggested that this pathway likely plays a functional role in soybean response to drought at the germination stage. Previous studies reported that genes involved in phenylpropanoid biosynthesis play crucial roles in response to salt stress (Cao et al., 2020; Hu et al., 2022). A total of 44 genes in the phenylpropanoid metabolism and biosynthesis pathway were differentially expressed in response to drought stress in maize at different developmental stages (Liu et al., 2021). Additionally, KEGG enrichment analysis of DEGs in key modules suggested that genes involved in plant hormone signal transduction, isoflavonoid biosynthesis, and the MAPK signaling pathway may also play crucial roles in response to drought stress. Consistent with our findings, ten key drought-tolerant genes were identified through an integrative data-driven feature engineering pipeline, and KEGG enrichment showed that the secondary metabolite synthesis pathway plays a crucial role in soybean drought resistance (Kao et al., 2025). Moreover, the research reported that genes related to hormone signaling are critical for drought stress response in maize (Liu et al., 2021), and another study reported that drought stress regulated the synthesis of flavonoids by several key genes, viz. *CHS*, *F3H*, *DFR*, and *ANS* (Li et al., 2022a), and that in turn enhances drought tolerance in soybean.

The MAPK signaling pathway is one of the significant conservative signal transduction pathways when plants convert extracellular drought stress signals into intracellular ones (Opdenakker et al., 2012). It has been reported that the MAPK signaling pathway is related to the drought tolerance of soybean seedlings (Xuan et al., 2022). Of 40 hub genes, two genes (*Glyma.13G229300* and *Glyma.08G188300*) were related to abscisic acid (ABA) signaling. It is well recorded that ABA participates in numerous plant abiotic stress responses, particularly in drought stress (Tae-Houn and Kim, 2014). When plants experience drought stress, ABA levels rapidly accumulate. ABA is then perceived and bound by its receptors (PYR/PYL/RCAR family proteins), which inactivate type 2C protein phosphatases (PP2Cs). This inhibition releases SnRK2 kinases from suppression, allowing their activation. The activated SnRK2s phosphorylate downstream ABA-responsive transcription factors (TFs), such as ABA-responsive element-binding proteins (AREBs)/ABRE-binding factors (ABFs), ultimately enhancing the plant’s drought resistance (Miyazono et al., 2009). Previously, most of the *GmSnRK2* genes could respond to drought stress after drought stress treatment (Shen et al., 2022). Similarly, in our current study, the expression level of *Glyma.08G188300* was upregulated both in DT and DS after 24h of drought treatment (**Supplementary Figure S10**). *PYL11* and *PYL12*, as ABA receptors, are highly expressed only in mature and dry seeds and have a beneficial effect on ABA-mediated seed germination (Zhao et al., 2020). In this study, the *Glyma.13G229300* was upregulated at 12h and 24h after drought treatment both in DT and DS (**Supplementary Figure S10**). These results suggest that these two genes function as positive regulators of drought stress response, likely by enhancing ABA signaling to improve plant drought tolerance.

Transcriptional factors have been designated master regulators of abiotic stresses, including drought (Wang et al., 2016). The NF-YA, NAC, GRAS, and bZIP families play an important role in drought stress response. For instance, overexpression of *GmNFYA13* (Ma et al., 2020a), *GmNFYA5* (Ma et al., 2020b), *GmNAC8* (Yang et al., 2020), and *GmGRAS37* (Wang et al., 2020) improved drought tolerance in soybean. In contrast, *GmbZIP15* negatively regulates salt and drought tolerance in soybean (Zhang et al., 2020). GmNF-YC14, as a positive regulator of drought tolerance in soybean, can interact with GmNF-YB2 and GmNF-YA16 to form a functional NF-Y transcription complex. This complex modulates the ABA signaling pathway by regulating PYR/PYL receptor genes, thereby enhancing drought stress resistance in plants (Yu et al., 2021). In this study, the expression levels of *Glyma.17G051400*, *Glyma.04G231400*, *Glyma.09G052800*, and *Glyma.13G124900* continued to increase after drought treatment. *Glyma.12G091200* was upregulated at 12h and then downregulated at 24h after drought treatment (**Supplementary Figure S10**). These findings suggest that the genes identified in the present study are closely associated with soybean drought tolerance. Further, the key genes identified in this study represent valuable genetic resources and potential targets for molecular breeding, offering promising avenues for developing drought-resistant soybean cultivars through genetic engineering approaches. However, whether these candidate genes, plus the priority candidate genes, do have functions in soybean drought tolerance needs further experimental verification. So, the functional validation of these hub genes will be key work in the future, which will provide new insights to better understand the molecular mechanism of drought tolerance at the germination stage in soybean. Additionally, drought tolerance is a complex trait, which is controlled by unequal polygenes and is susceptible to environmental influences. The QTL-by-environment interaction and the QTL-by-QTL interaction should also be detected in the future.

## Data Availability

The datasets presented in this study can be found in online repositories. The names of the repository/repositories and accession number(s) can be found in the article/**Supplementary Material**.
