# Supplementary material for: Genome-wide association study and transcriptome analysis reveal candidate genes related to drought stress in the germination stage of soybean
Source: Front Plant Sci. 2025 Jul 30;16:1621869. doi: 10.3389/fpls.2025.1621869 (PMC12343491; doi:10.3389/fpls.2025.1621869)
Supplement: Supplementary file 1 [file DataSheet1.docx]

Supplementary Material


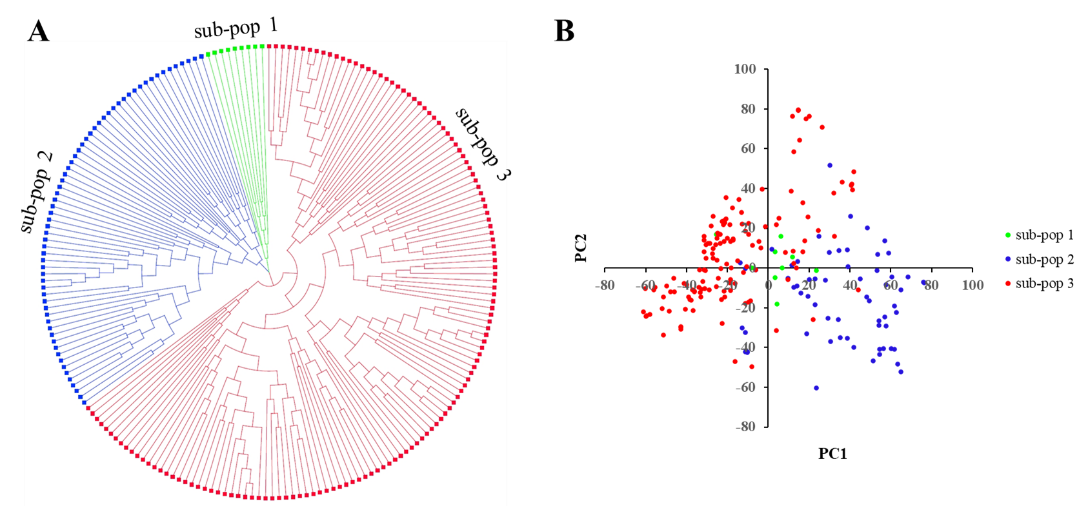


**Supplementary Figure 1.** Population structure in 207 soybean accessions.

A, Neighbour-joining tree of the 207 accessions using Nei’s genetic distance. B, Scatter plot of the 207 soybean accessions using the principal component analysis (PCA) with the first two principal components.


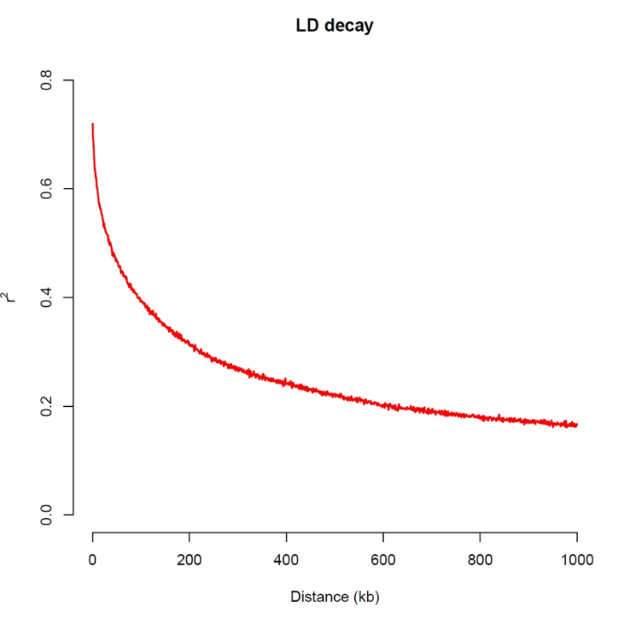


**Supplementary Figure 2.** Linkage disequilibrium (LD) decay distance across 20 chromosomes in 207 soybean accessions.


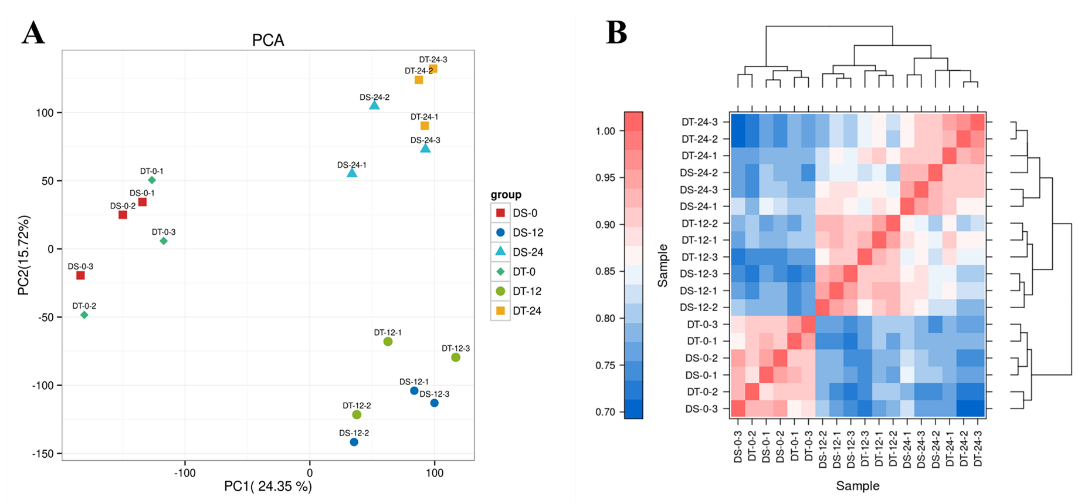


**Supplementary Figure 3.** Global gene expression profiling under drought stress at different time points. (A) PCA of the RNA-Seq data from three time points under drought stress in drought tolerance and drought sensitive varieties. (B) Spearman correlation coefficient analysis of RNA-seq data.


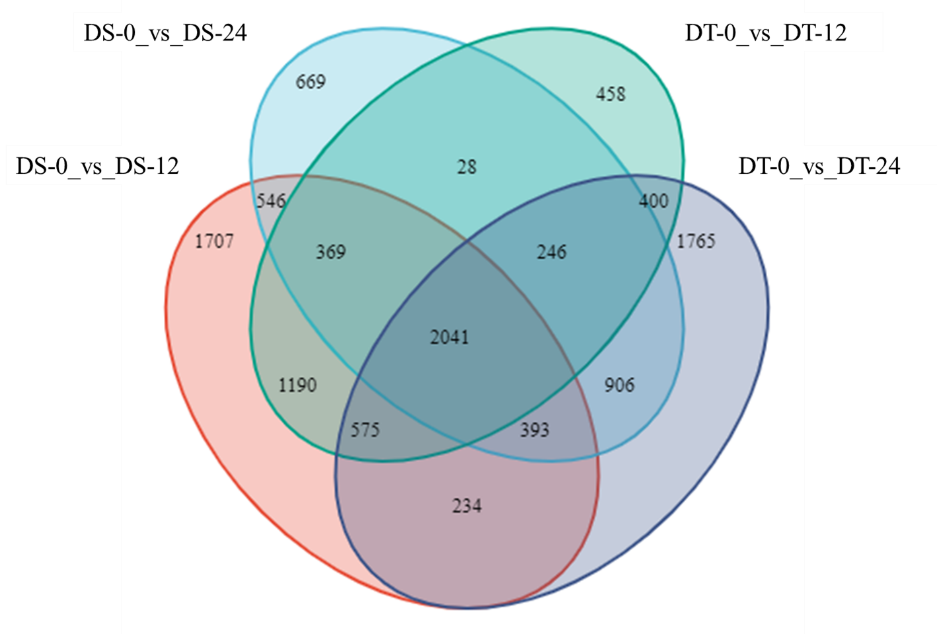


**Supplementary Figure 4.** Venn diagrams of DEGs among different samples.


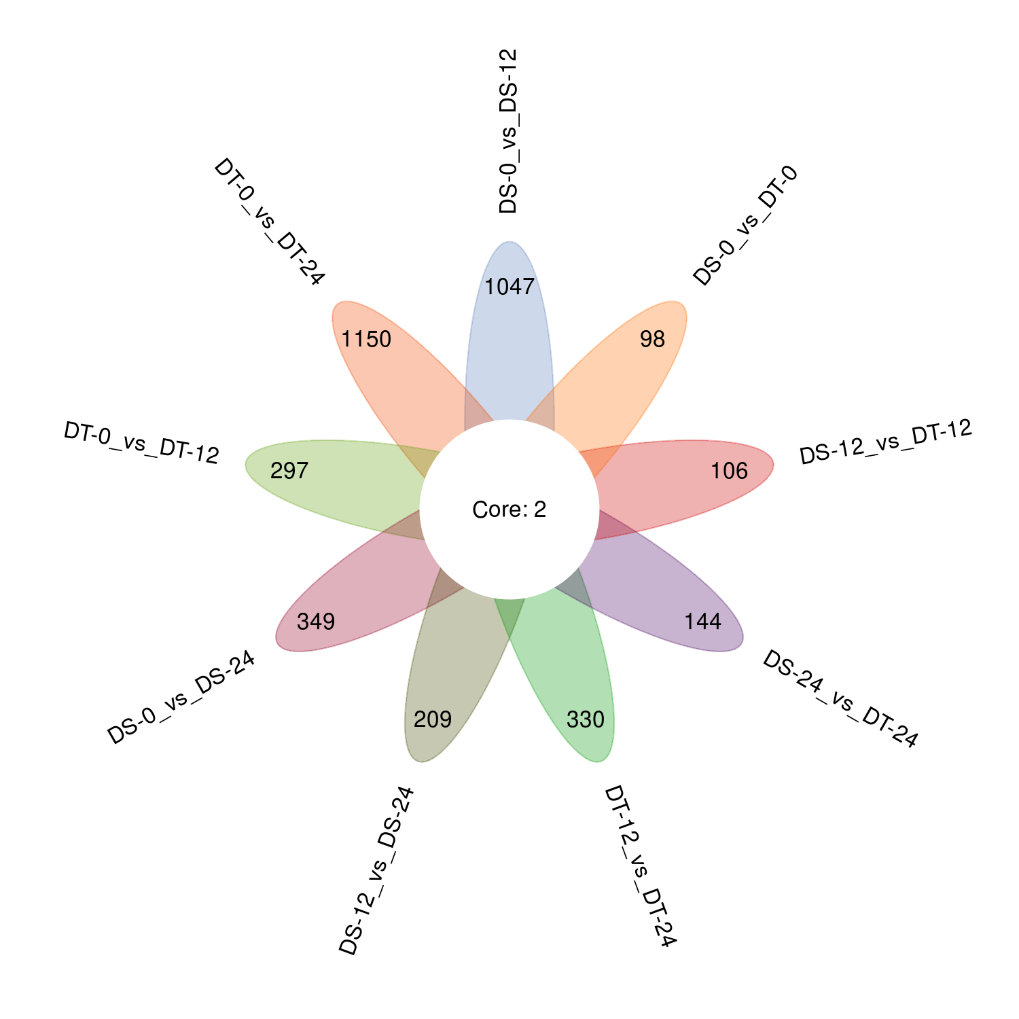


**Supplementary Figure 5.** Visualization of DEG intersections using a petal plot.


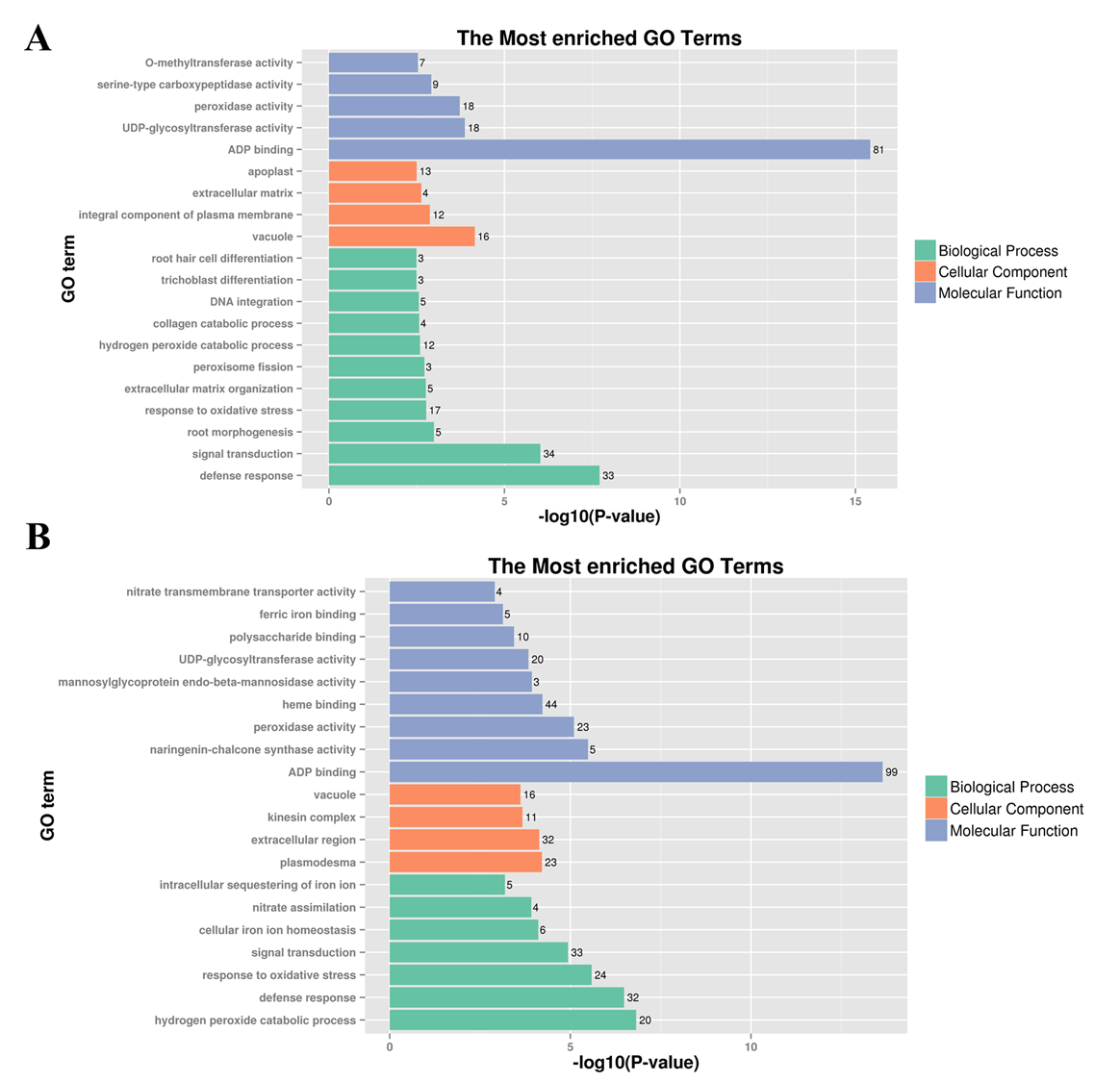


**Supplementary Figure 6.** GO enrichment analysis for DEGs between DS and DT at two time points.

(A) and (B), the top 20 GO terms with the highest significance after 12 h of drought treatment and after 24 h, respectively.


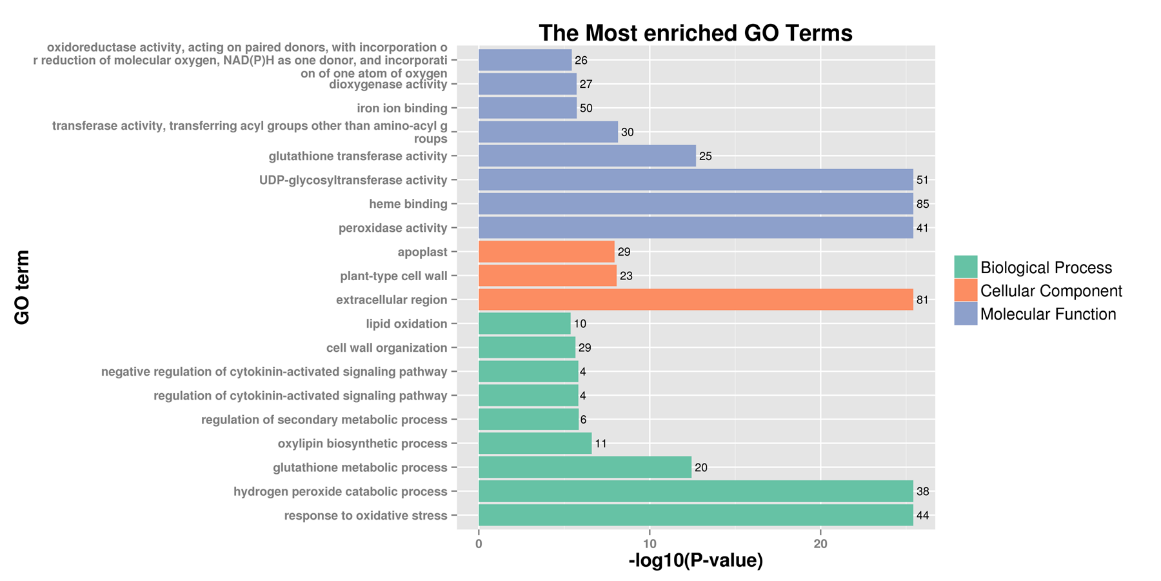


**Supplementary Figure 7.** GO enrichment analysis for drought-responsive DEGs shared by DS and DT at two time points. The vertical axis represents the GO term. The horizontal axis represents the value of -log10(P-value). The numbers on the bar represent the number of genes enriched in corresponding GO term.


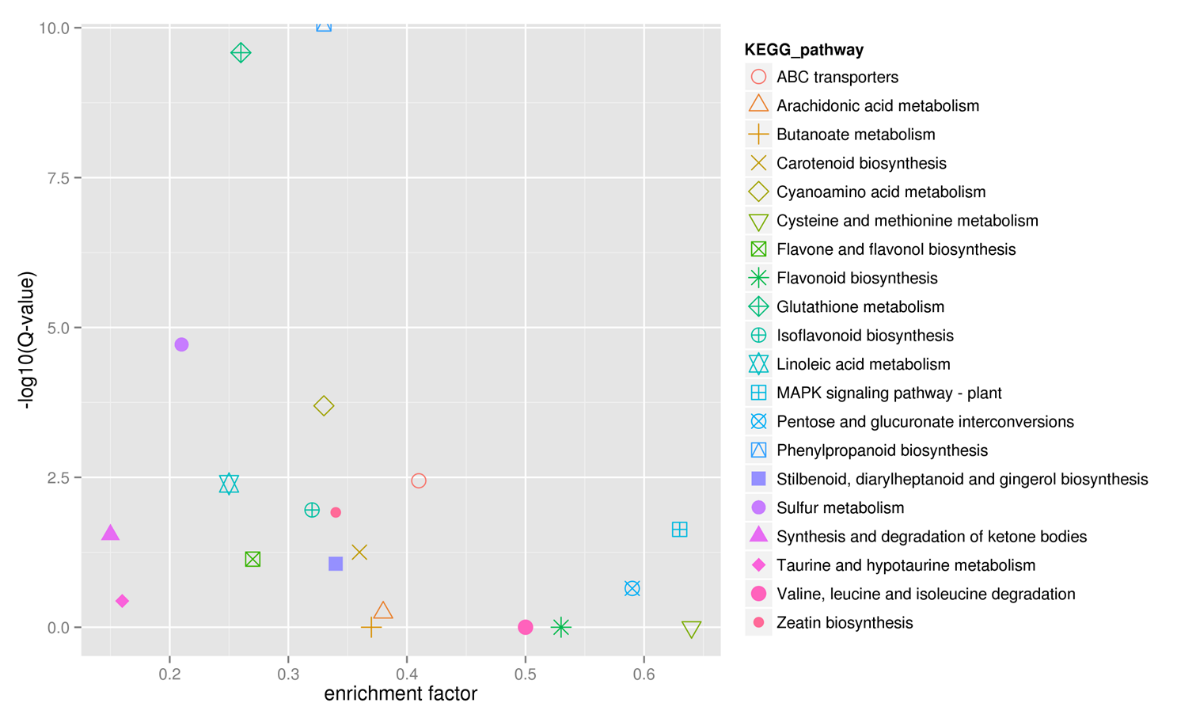


**Supplementary Figure 8.** KEGG enrichment analysis for drought-responsive DEGs shared by DS and DT at two time points. The vertical axis represents the value of -log10(Q-value). The horizontal axis represents the enrichment score, indicating the degree of enrichment of a gene set in a specific pathway. The higher the score, the higher the degree of enrichment.


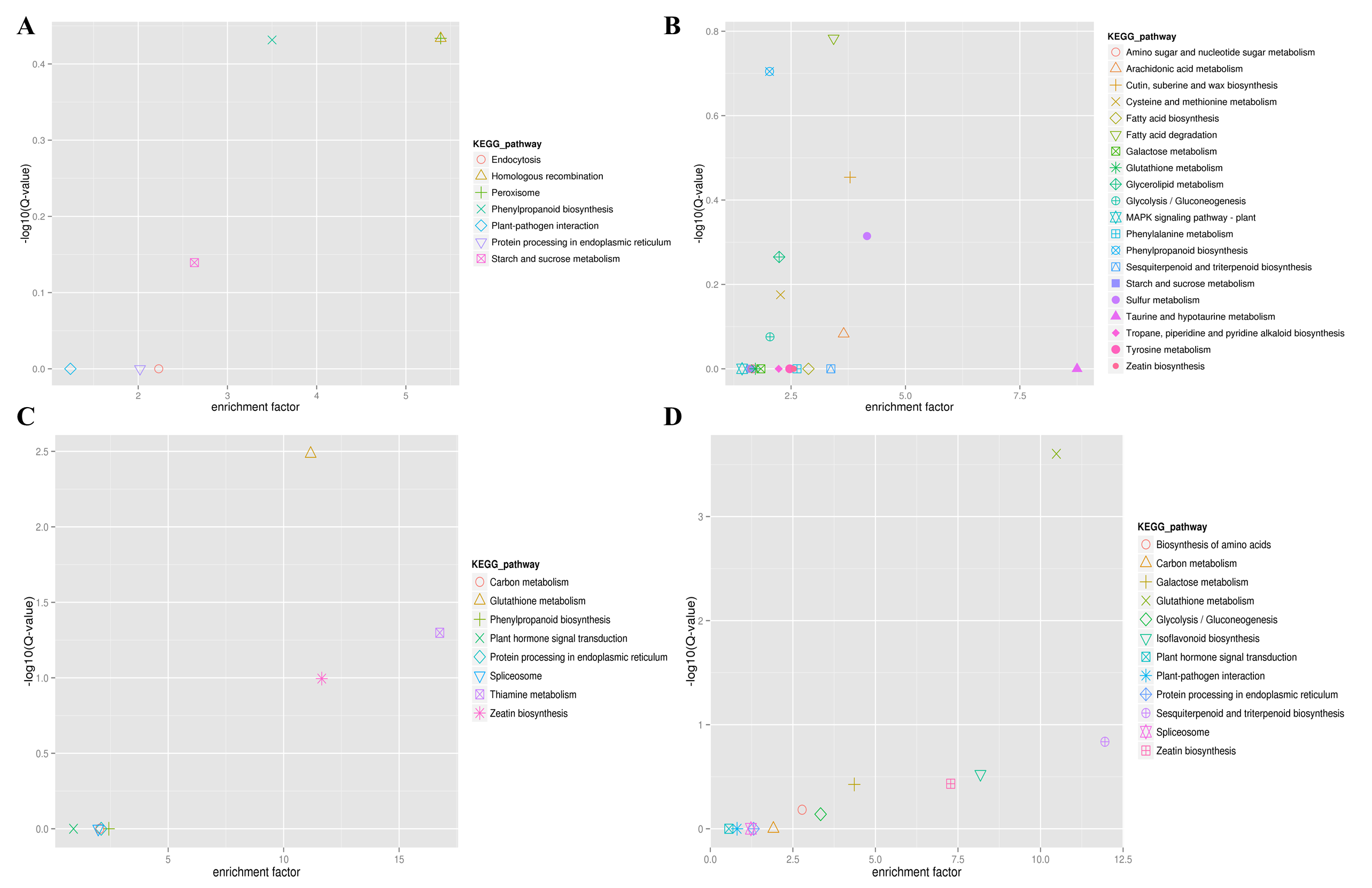


**Supplementary Figure 9.** KEGG enrichment analysis of selected four key modules. A-D represent the KEGG pathway enrichment in the paleturquoise, darkolivegreen, skyblue2, and mediumorchid modules, respectively. The vertical axis represents the value of -log10(Q-value). The horizontal axis represents the enrichment score, indicating the degree of enrichment of a gene set in a specific pathway. The higher the score, the higher the degree of enrichment.


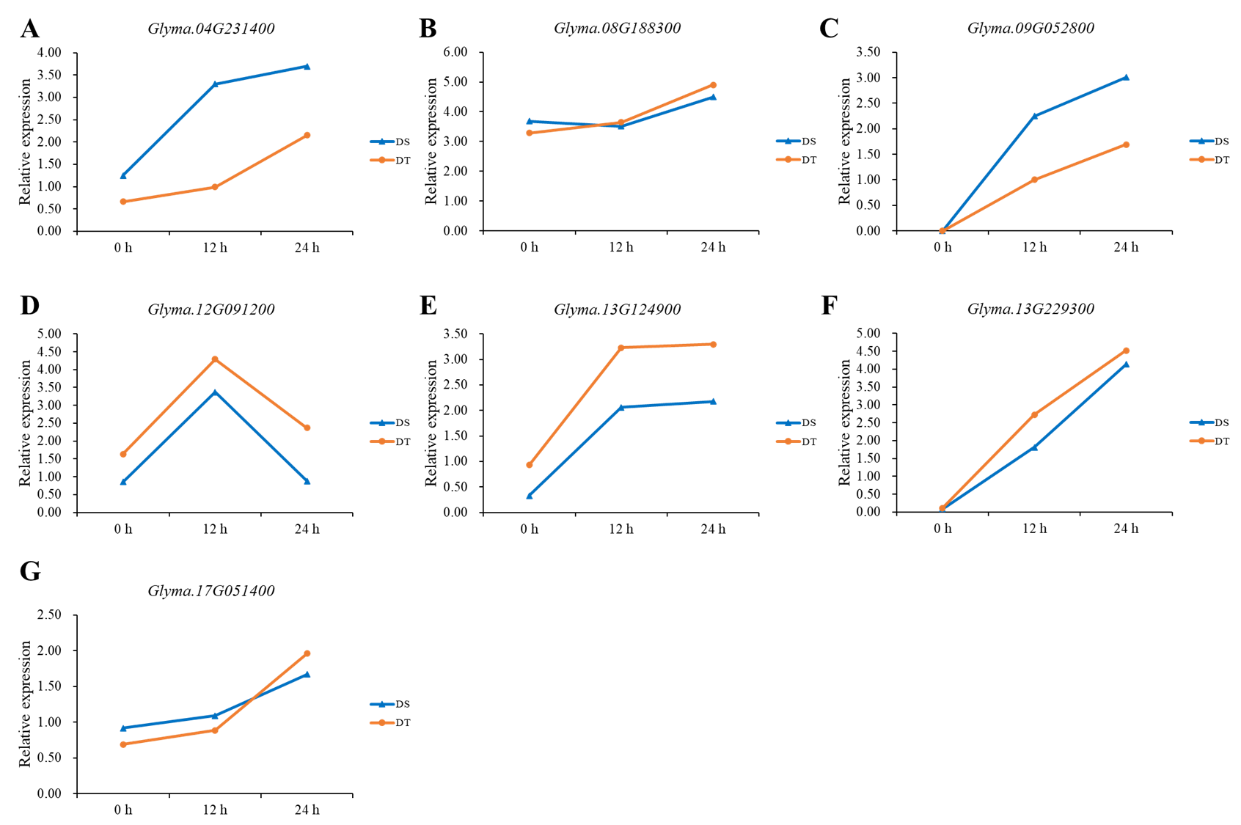


**Supplementary Figure 10.** The expression analysis of seven hub genes in DT (high drought-tolerant soybean accession) and DS (high drought-sensitive soybean accession) after 0, 12, and 24 h of drought treatment based on the RNA-seq data. The FPKM value was normalized by log2^(FPKM+1)^. A-G represent the expression patterns of *Glyma.04G231400*, *Glyma.08G188300*, *Glyma.09G052800*, *Glyma.12G091200*, *Glyma.13G124900*, *Glyma.13G229300* and *Glyma.17G051400*, respectively.
